# Case of Imperforate Vagina—Operation—Death—Dissection—Remarks

**Published:** 1830-10-01

**Authors:** 


### LOWESTOFT INFIRMARY.

Case of Imperforate Vagina—Operation—Death, with the Dissection.

[Reported by Mr. W. C. Worthington.]

Frances Baker, æt. 14, of a precocious appearance, was admitted a patient of the Lowestoft Dispensary on the 7th July, complaining of violent paroxysms of pain in the abdomen and loins, shooting down the thighs, and with occasional difficulty in passing her urine. In the hypogastric region was discovered a circumscribed elastic swelling, rising above the brim of the pelvis, which she described as having progressively increased for the last three months. From the situation and feel of the tumour Mr. W. was led to suspect some uterine disease existed, and upon endeavouring to make an examination *per vaginam*, to satisfy himself upon that point, he was surprised at an irresistible impediment to the introduction of the finger. On a more accurate investigation, the orifice of the vagina was found to be preternaturally and effectually closed by a firm adhesion of the parts.

Medical treatment having failed to afford her any relief, it became apparent that the swelling and pain were owing to the uterus being distended by the retained menstrual fluid. This opinion was further confirmed by an examination *per rectum*, through which a tumour was perceptible, possessing a distinct fluctuation, and descending towards the perinæum.

The operation consisted in carefully dividing with a scalpel a dense cellular structure, of about half an inch in thickness, situated at the orifice of the vagina. A thin membranous expansion being left was then punctured with a lancet, which gave exit to about a pound of dark-coloured fluid. The swelling immediately disappeared and the girl expressed herself relieved. A sponge-tent, well oiled, was inserted, and retained between the divided parts.

The third day after the operation, severe pain in the abdomen, with exquisite tenderness supervened, together with excessive gastric irritation. Notwithstanding a strict antiphlogistic plan of treatment was adopted, the patient died the following morning.

On examination after death, the peritoneum was found to have been generally affected with inflammation; various gangrenous spots were presented to view, also a considerable quantity of lymph was effused, causing adhesion of the convolutions of the bowels. The uterus was nearly of its ordinary size, but the vagina was dilated into a pouch, contracted towards its orifice, capable of holding a pint and a half of fluid. Its parietes were much thickened, and of a semi-cartilaginous structure.

Professor Langenbec* relates a similar case, in which death took place the fifth day after the operation, and attributes the tendency to inflammation to the long retention of the menses. He, therefore, very judiciously advises the operation never to be delayed when the true nature of the complaint is discovered; an opinion worthy of attention, inasmuch as some surgeons have considered all interference as improper, until such time as the tumour shall have attained a large size, grounding their opinions upon the principle, that it may then be punctured with more facility.

If the professor's views on the subject be correct, the practice of an early operation in cases of imperforate vaginæ cannot be too strongly enforced, and the reporter cannot but be inclined to believe that the unfortunate issue of this case has contributed to verify the truth of them.

---

* Langenbec's Bibliothek, Vol. IV. pt. 3.

